# Supplementary material for: Cohesin prevents cross-domain gene coactivation
Source: Nat Genet. 2024 Jul 24;56(8):1654–64. doi: 10.1038/s41588-024-01852-1 (PMC11319207; doi:10.1038/s41588-024-01852-1)

nature genetics

Supplementary information https://doi.org/10.1038/s41588-024-01852-1

Cohesin prevents cross-domain gene coactivation

In the format provided by the authors and unedited

The following image illustrates our gating strategy for flow cytometry analysis related to Extended Data Fig.7k.

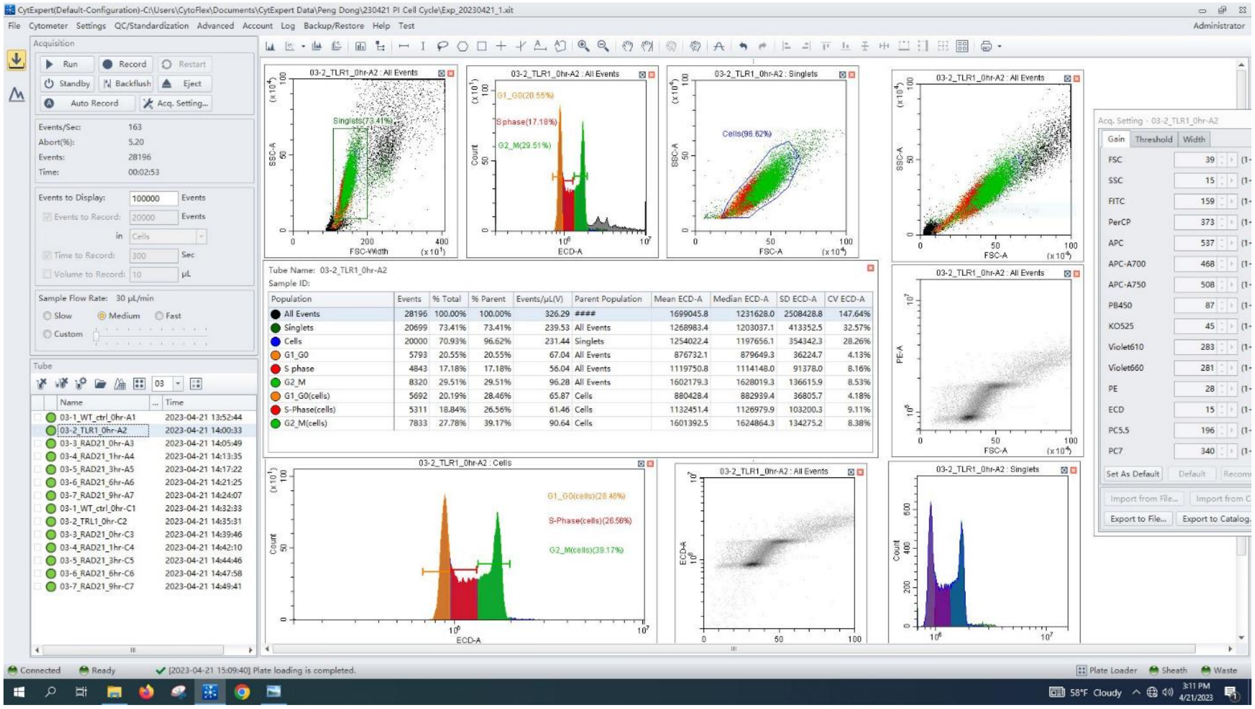

Supplement: Supplementary file 1 — Supplementary information. [file 41588_2024_1852_MOESM1_ESM.pdf]
