# Supplementary material for: Cohesin prevents cross-domain gene coactivation
Source: Nat Genet. 2024 Jul 24;56(8):1654–64. doi: 10.1038/s41588-024-01852-1 (PMC11319207; doi:10.1038/s41588-024-01852-1)
Supplement: Supplementary file 2 — Reporting Summary [file 41588_2024_1852_MOESM2_ESM.pdf]

nature portfolio

Corresponding author(s): Zhe J. Liu

Last updated by author(s): 02/22/2023

Reporting Summary

Nature Portfolio wishes to improve the reproducibility of the work that we publish. This form provides structure for consistency and transparency in reporting. For further information on Nature Portfolio policies, see our Editorial Policies and the Editorial Policy Checklist.

Statistics

For all statistical analyses, confirm that the following items are present in the figure legend, table legend, main text, or Methods section.

n/a	Confirmed	
☐	☒	The exact sample size (n) for each experimental group/condition, given as a discrete number and unit of measurement
☐	☒	A statement on whether measurements were taken from distinct samples or whether the same sample was measured repeatedly
☐	☒	The statistical test(s) used AND whether they are one- or two-sided<br>*Only common tests should be described solely by name; describe more complex techniques in the Methods section.*
☐	☒	A description of all covariates tested
☐	☒	A description of any assumptions or corrections, such as tests of normality and adjustment for multiple comparisons
☐	☒	A full description of the statistical parameters including central tendency (e.g. means) or other basic estimates (e.g. regression coefficient) AND variation (e.g. standard deviation) or associated estimates of uncertainty (e.g. confidence intervals)
☐	☒	For null hypothesis testing, the test statistic (e.g. F, t, r) with confidence intervals, effect sizes, degrees of freedom and P value noted<br>*Give P values as exact values whenever suitable.*
☒	☐	For Bayesian analysis, information on the choice of priors and Markov chain Monte Carlo settings
☒	☐	For hierarchical and complex designs, identification of the appropriate level for tests and full reporting of outcomes
☐	☒	Estimates of effect sizes (e.g. Cohen's d, Pearson's r), indicating how they were calculated

Our web collection on statistics for biologists contains articles on many of the points above.

Software and code

Policy information about availability of computer code

Data collection	All data collection software/codes are published
Data analysis	The visualization software (VVD-viewer) for spatial genome imaging data is available here: https://github.com/takashi310/VVD_Viewer

For manuscripts utilizing custom algorithms or software that are central to the research but not yet described in published literature, software must be made available to editors and reviewers. We strongly encourage code deposition in a community repository (e.g. GitHub). See the Nature Portfolio guidelines for submitting code & software for further information.

Data

Policy information about availability of data

All manuscripts must include a data availability statement. This statement should provide the following information, where applicable:

- Accession codes, unique identifiers, or web links for publicly available datasets
- A description of any restrictions on data availability
- For clinical datasets or third party data, please ensure that the statement adheres to our policy

Sequencing data that support the findings of this study will be deposited in NCBI-GEO database. Accession codes will be available before publication.

Human research participants

Policy information about studies involving human research participants and Sex and Gender in Research.

Reporting on sex and gender	N/A
Population characteristics	N/A
Recruitment	N/A
Ethics oversight	N/A

Note that full information on the approval of the study protocol must also be provided in the manuscript.

Field-specific reporting

Please select the one below that is the best fit for your research. If you are not sure, read the appropriate sections before making your selection.

- [x] Life sciences
- [ ] Behavioural & social sciences
- [ ] Ecological, evolutionary & environmental sciences

For a reference copy of the document with all sections, see nature.com/documents/nr-reporting-summary-flat.pdf

Life sciences study design

All studies must disclose on these points even when the disclosure is negative.

Sample size	For genomic analysis, > 200,000 pairwise correlations of 776 ACDs were performed for each condition; for spatial genome imaging, >1000 cells were analyzed for each condition; for single molecule analysis, > 10,000 trajectories were analyzed for each condition.
Data exclusions	No data were excluded from this study.
Replication	High-rougthput sequencing data (scATAC-seq and Smart-SCRB) were repeated at least twice with biological replicates. All imaging data were repeated at least three times with biological replicates.
Randomization	Samples from different perturbation conditions were grouped and analyzed without a particular order.
Blinding	Data were processed with automated data analysis pipelines without human interference. No blinding is needed.

Reporting for specific materials, systems and methods

We require information from authors about some types of materials, experimental systems and methods used in many studies. Here, indicate whether each material, system or method listed is relevant to your study. If you are not sure if a list item applies to your research, read the appropriate section before selecting a response.

Materials & experimental systems

n/a	Involved in the study	
[ ]	[x]	Antibodies
[ ]	[x]	Eukaryotic cell lines
[x]	[ ]	Palaeontology and archaeology
[x]	[ ]	Animals and other organisms
[x]	[ ]	Clinical data
[x]	[ ]	Dual use research of concern

Methods

n/a	Involved in the study	
[x]	[ ]	ChIP-seq
[ ]	[x]	Flow cytometry
[x]	[ ]	MRI-based neuroimaging

Antibodies

Antibodies used	anti-RAD21(D213) rabbit polyclonal antibody (Cell Signaling, Cat. 4321);anti-MED1(CRSP1/TRAP220) rabbit polyclonal antibody (Bethyl Laboratories Inc, Cat. A300-793A), anti-MED6 Rabbit polyclonal antibody (Abcam, Cat. ab220110), anti-BRD4 (E8V7I) Rabbit monoclonal antibody (Cell Signaling, Cat. 54615), anti-TBP Rabbit polyclonal antibody (Cell Signaling, Cat. 8515), anti-OCT4 rabbit polyclonal antibody (Abcam, Cat. ab19857), Anti-Histone H2A.Z antibody [EPR18090] (Abcam, Cat. ab188314) and anti- -tubulin (11H10) rabbit monoclonal antibody (Cell Signaling, Cat. 2125).

Validation	anti-RAD21(D213) rabbit polyclonal antibody (Cell Signaling, Cat. 4321); application: Western blot; validation: knockout<br>anti-MED1(CRSP1/TRAP220) rabbit polyclonal antibody (Bethyl Laboratories Inc, Cat. A300-793A); application: Western blot; validation: HaloTag knock-in band shift<br>anti-MED6 Rabbit polyclonal antibody (Abcam, Cat. ab220110); application: Western blot; validation: HaloTag knock-in band shift<br>anti-BRD4 (E8V7I) Rabbit monoclonal antibody (Cell Signaling, Cat. 54615); application: Western blot; validation: HaloTag knock-in band shift<br>anti-TBP Rabbit polyclonal antibody (Cell Signaling, Cat. 8515); application: Western blot; validation: overexpression of HaloTag TBP band shift<br>anti-OCT4 rabbit polyclonal antibody (Abcam, Cat. ab19857); application: Western blot; validation: overexpression of HaloTag Oct4 band shift<br>anti-Histone H2A.Z antibody [EPR18090] (Abcam, Cat. ab188314); application: Western blot; validation: overexpression of HaloTag Oct4 band shift<br>anti-tubulin (11H10) rabbit monoclonal antibody (Cell Signaling, Cat. 2125). application: Western blot; validation; western blots

Eukaryotic cell lines

Policy information about cell lines and Sex and Gender in Research

Cell line source(s)	JM8.N4 mouse embryonic stem cells (mESCs)
Authentication	4DN Consortium Recommended Cell Lines; https://www.4dnucleome.org/cell-lines/
Mycoplasma contamination	Negative for Mycoplasma contamination
Commonly misidentified lines (See ICLAC register)	*Name any commonly misidentified cell lines used in the study and provide a rationale for their use.*

Flow Cytometry

Plots

Confirm that:

- [x] The axis labels state the marker and fluorochrome used (e.g. CD4-FITC).
- [ ] The axis scales are clearly visible. Include numbers along axes only for bottom left plot of group (a 'group' is an analysis of identical markers).
- [ ] All plots are contour plots with outliers or pseudocolor plots.
- [ ] A numerical value for number of cells or percentage (with statistics) is provided.

Methodology

Sample preparation	Cell cycle analysis was performed by propidium iodide (PI) staining following the protocols from the propidium iodide flow cytometry kit (Abcam, Cat. ab139418).
Instrument	Beckman Coulter CytoFLEX S with 4 lasers (405nm, 488nm, 561nm, 638nm) (Beckman Coulter)
Software	CytExpert Software v2.3 (Beckman Coulter)
Cell population abundance	All samples were acquired for 5 mins at a sampling rate of 30µl/min or up to 15,000 cells.
Gating strategy	SSC-A versus FSC-W was used for initial gating of singlet cells followed by SSC-A versus FSC-A to further define cellular events. An event count versus 561-610/20 histogram plot was used to determine the percentage of cells in G1, S or G2/M phases of the cell cycle.

- [ ] Tick this box to confirm that a figure exemplifying the gating strategy is provided in the Supplementary Information.
