# Supplementary material for: Cohesin prevents cross-domain gene coactivation
Source: Nat Genet. 2024 Jul 24;56(8):1654–64. doi: 10.1038/s41588-024-01852-1 (PMC11319207; doi:10.1038/s41588-024-01852-1)
Supplement: Supplementary file 20 — Unprocessed gels and blots. [file 41588_2024_1852_MOESM20_ESM.pdf]

Related to Extended Data Figure 7c

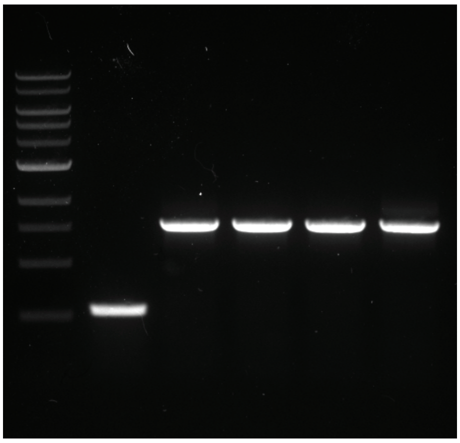

Related to Extended Data Figure 7d

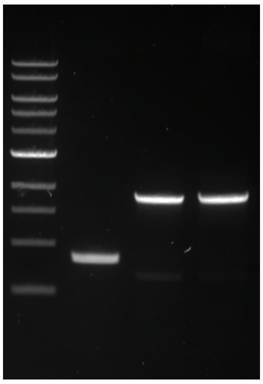

Related to Extended Data Figure 7e

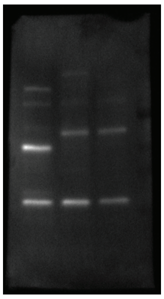

Anti-MED6

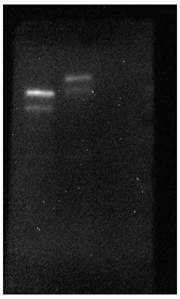

Anti-RAD21

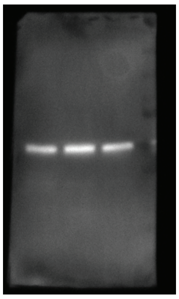

Anti-α-tubulin

Related to Extended Data Figure 7f

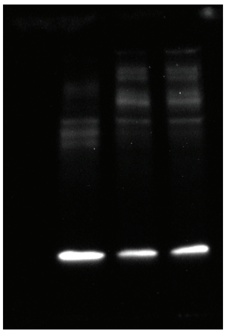

Anti-MED1

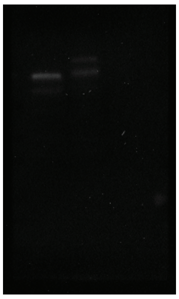

Anti-RAD21

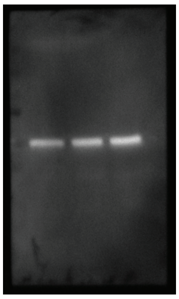

Anti-α-tubulin

Related to Extended Data Figure 7g

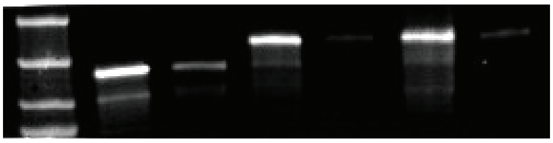

Anti-RAD21

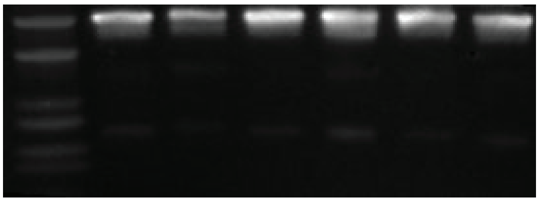

Anti-α-tubulin

Related to Extended Data Figure 7h

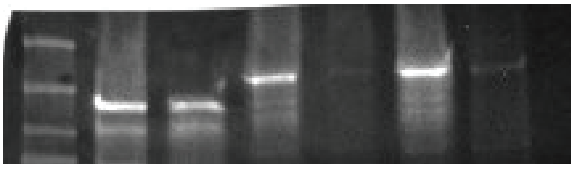

Anti-RAD21

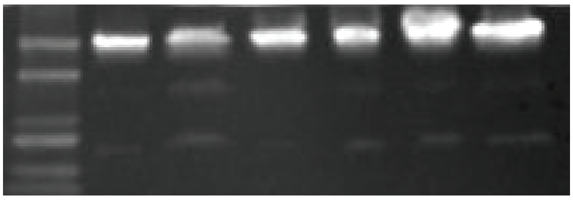

Anti-α-tubulin
