# Supplementary material for: Cohesin prevents cross-domain gene coactivation
Source: Nat Genet. 2024 Jul 24;56(8):1654–64. doi: 10.1038/s41588-024-01852-1 (PMC11319207; doi:10.1038/s41588-024-01852-1)
Supplement: Supplementary file 22 — Unprocessed gels and blots. [file 41588_2024_1852_MOESM22_ESM.pdf]

Related to Extended Data Figure 9a

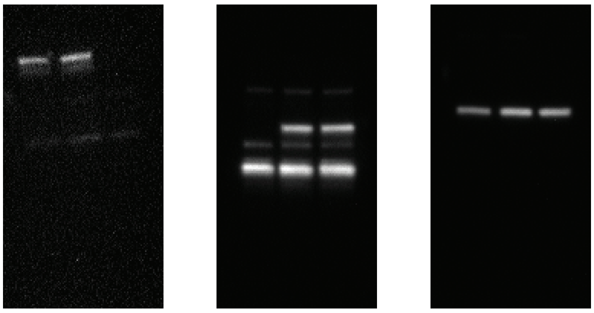

Anti-RAD21 Anti-OCT4 Anti-α-tubulin

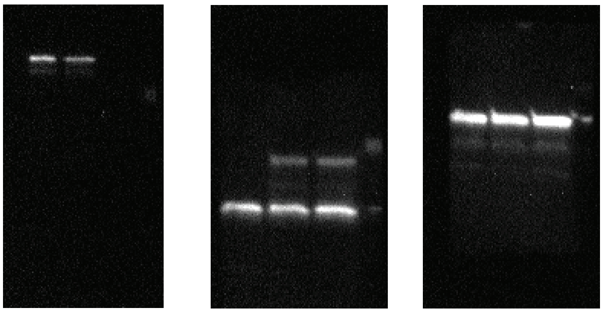

Anti-RAD21 Anti-mTBP Anti-α-tubulin

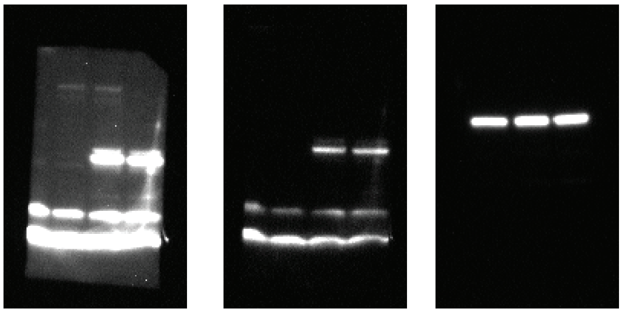

Anti-RAD21* Anti-H2A.Z Anti-α-tubulin

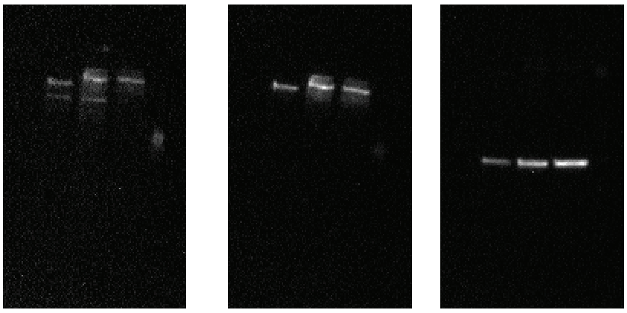

Anti-RAD21[#] Anti-BRD4 Anti-α-tubulin

Note: *blotted on the sample after stripping of H2A.Z antibody
[#]blotted on the sample after stripping of BRD4 antibody
